# Performance, Feeding Behavior and Immune Response in Nellore and Angus × Nellore Steers Fed Whole Shelled Corn Diets with or without Fiber

**DOI:** 10.3390/ani12192692

**Published:** 2022-10-06

**Authors:** Aline Castro Rodrigues, Priscilla Dutra Teixeira, Daniel Rume Casagrande, Ana Paula Peconick, Tamara Cristina Coelho, Pedro Veiga Rodrigues Paulino, Márcio Machado Ladeira

**Affiliations:** 1 Department of Animal Science, College of Animal Science and Veterinary Medicine, Universidade Federal de Lavras, Lavras 37200-900, Minas Gerais, Brazil; 2 Department of Veterinary Medicine, College of Animal Science and Veterinary Medicine, Universidade Federal de Lavras, Lavras 37200-900, Minas Gerais, Brazil; 3 Cargill Animal Nutrition, Campinas 13091-611, São Paulo, Brazil

**Keywords:** *Bos indicus*, crossbred, feedlot, haptoglobin

## Abstract

**Simple Summary:**

Grain diets provide a higher energy supply during the finishing phase, however, they can reduce rumen pH, leading to physiological stress in animals, affecting immune and metabolic systems and animal performance. To avoid this, an alternative is using fiber sources in low quantities. This study indicated that using fiber sources increases DMI and performance in whole shelled corn diets, and greater DMI increases haptoglobin blood concentration in low fiber diets.

**Abstract:**

This study aimed to evaluate performance, to evaluate performance, carcass traits, feeding behavior, and humoral immune response in Nellore and Angus × Nellore steers fed whole shelled corn diets. Sixteen Nellore and 16 Angus × Nellore steers with 353 ± 25.3 kg were randomly assigned into 2 feeding groups, whole shelled corn without forage (WSC) or whole shelled corn and sugarcane bagasse (WSCB). The data were analyzed using the MIXED procedure of SAS (SAS Inst. Inc., Cary, NC, USA). Angus × Nellore steers had greater final BW, DMI, ADG, and subcutaneous fat thickness than Nellore. Moreover, Nellore steers had lower neutral detergent fiber (NDF) intake but spent more time ruminating and had a greater rumination rate of NDF. Regarding the diets, steers fed the WSCB had greater DMI and spent more time chewing and ruminating. Nellore × Angus steers had a greater haptoglobin concentration. The concentration of D-Lactate tended to be greater in the Nellore steers regardless of diet. In conclusion, the use of sugarcane bagasse in WSC diets increases DMI and ADG without affecting feed efficiency or the carcass traits of the steers. Although Angus × Nellore steers have increased haptoglobin concentration, this effect was not enough to reduce the performance of these animals.

## 1. Introduction

Feedlot diets have changed in Brazil, with the increase of concentrate levels in their composition. The usual level of concentrate in Brazilian feedlot diets is between 71% to 90% [1]. Additionally, the authors reported that 5.56% of Brazilian feedlots used whole shelled corn in their diets. Diets with a high proportion of concentrate, or even diets without forage, began to be widely used in Brazilian feedlots over the past decade.

In addition, starch is the main component of a beef cattle diet, as it provides higher energy supply during the finishing phase [2]. However, increased starch supply to the rumen can cause organic acid accumulation [3] and reduced rumen pH due to rapid and excessive fermentation [4], plus shorter rumination time [5]. In addition, high starch contents can lead to physiological stress in the animal [6] and possibly acidosis [7]. Cattle with acidosis can absorb free lipopolysaccharides [8,9], and this absorption results in the production of proinflammatory cytokines and acute-phase proteins [10,11,12], affecting immune and metabolic responses [13,14] and animal performance [15,16,17]. Adding fiber sources is an alternative to avoid this effect from high grain diets. Fiber sources can improve the rumen environment and reduce free lipopolysaccharide production [18]. In addition, fiber sources may increase dry matter intake (DMI) and, consequently, average daily gain (ADG) [19,20].

According to Krehbiel et al. [21], *Bos taurus* has greater dry matter intake in relation to maintenance requirements than *Bos indicus* when fed concentrate-based diets and, therefore, has better performance and is more efficient. In addition, *Bos* *indicus* develops more acidosis problems when fed high-starch diets, resulting in lower performance [22]. Carvalho et al. [2], evaluating the use of whole shelled corn diets without forage, found that purebred Angus had better performance than Nellore. In addition, Cunha et al. [23] found that Angus × Nellore bulls showed greater average daily gain and feed efficiency than Nellore bulls feeding whole shelled corn diets.

This study hypothesizes that steers fed whole shelled corn with sugarcane bagasse have greater DMI and ADG and may have better immunological status than steers fed the diet without the bagasse. An additional hypothesis is that Angus × Nellore steers fed whole shelled corn diets have greater ADG, feed efficiency, and marbling scores than Nellore steers. To test this hypothesis, the objective of this study is to evaluate the performance, carcass traits, feeding behavior, and blood concentration of tumor factor necrosis factor alpha (TNF-α) and haptoglobin in Nellore and Angus × Nellore steers fed whole shelled corn diets with or without fiber source.

## 2. Materials and Methods

The general temperature during the feedlot was 23.3° with a humidity of 64%. Meteorological data were obtained from a weather station located at the Federal University of Lavras.

### 2.1. Experimental Design, Animals, and Diets

Sixteen Nellore and 16 Angus × Nellore steers with an initial live weight of 353 kg ± 25.3 kg were housed in individual pens in a completely randomized design in a 2 × 2 factorial arrangement, consisting of the following treatments: Nellore fed a whole shelled corn diet (WSC); Nellore fed a whole shelled corn with sugarcane bagasse diet (WSCB); Angus × Nellore fed the WSC diet; Angus × Nellore fed the WSCB diet. The experimental diets were offered ad libitum but were adjusted not to exceed 5% of orts, three times daily (08:00 am, 1:00 pm, and 4:00 pm). One experimental diet consisted of 80% whole shelled corn and 20% soybean meal and mineral-based pelleted supplement (Table 1). The other diet (WSCB) had 74% whole shelled corn, the same 20% pelleted supplement, and 6% sugarcane bagasse.

### 2.2. Performance, Behavior Study, and Chemical Composition of Experimental Diets

The steers were given 20 days to adapt to the facilities and the diets (step-up protocol), followed by 96 days of the experimental period. To measure ADG, steers were individually weighed at the beginning and the end of the experimental period, after fasting for 16 h (feed and water restrictions) before feeding the animals, using a digital scale (MGR-3000; Toledo do Brasil Ltd.a., São Bernardo do Campo, Brazil). The intake was individually measured, where the feed offered was recorded daily in the morning, and the orts on the following day were weighed against first feed to calculate DMI.

Variation in DMI (% of DM) was calculated using the difference in DMI between 2 consecutive days, according to Bevans et al. [24]. Weekly (fifteen weeks in total) samples of feed, orts, and ingredients of the diets were collected, and a composite sample of each was made. These samples were pre-dried in a forced-air circulation oven at 55 °C for 72 h and then ground in a grinder with a 1-mm mesh sieve. Chemical analyses of the diets were performed according to the Association of Official Analytical Chemists (AOAC) [25]. The NDF was measured according to Van Soest et al. [26], without sodium sulfite and with alpha-amylase, and the non-fiber carbohydrates were calculated according to Sniffen et al. [27]. Metabolizable energy (ME) was determined to convert the value of TDN to ME, according to NASEM (2016), using the data from the digestibility study [28].

The fractional passage rate of the digest was estimated by the disappearance of chromium concentration in the feces after an oral dose of 25 g of chromium oxide. Fecal samples were taken from each animal at 12, 24, 36, 48, and 84 h after the final administration of the marker. The passage rate (kp) was evaluated by fitting a simple linear regression equation of chromium concentrations over time. The chromium content in the fecal samples was analyzed according to Souza et al. [29]. Chromium quantification in the solutions was performed by atomic absorption spectrophotometry in the Leaf Analysis Laboratory of Federal University of Lavras (Lavras, MG).

Feeding behavior was also analyzed by a visual observation technique, by six trained people, over a continuous 24 h period on day 50 of the experiment. In this day, the general temperature on this day was 22.4° with a humidity of 64% and 0 mm rain. The observed behavior (feeding and ruminating) was recorded every 5 min. Time spent eating and time spent ruminating were used to calculate the time spent chewing, time spent eating/DMI and NDF intake, time spent ruminating/DMI and NDF intake, and time spent chewing/DMI and NDF intake [30]. All data were expressed in min kg^−1^ of DM and min kg^−1^ of NDF.

### 2.3. Blood Analyses

Blood samples were taken from the coccygeal vein to measure D-lactate, TNF-α, and haptoglobin levels on day 45. Blood samples were collected in Vacutainer tubes (Vacutainer, 10 mL; Becton Dickinson, Franklin Lakes, NJ, USA) containing sodium heparin for plasma collection to analyze TNF-α and haptoglobin, and without sodic heparin, to measure serum D-lactate.

After collection, blood samples were kept at room temperature for 30 to 180 min to form the clot and the complete collection of serum. For plasma, the samples were centrifuged at 2500× *g* for 30 min at 4 °C. Plasma and serum were separated and stored at −80 °C until analysis.

The TNF-α dose and haptoglobin were analyzed using a commercial kits (Cusabio Technology LLC, Houston, USA; Wuhan Fine Biotech Co., Wuhan, China, respectively), according to manufacturers’ instructions. The D-lactate levels were analyzed spectrophotometrically (Thermo Fisher Scientific, Waltham, MA, USA) using the commercial kit D-Lactate (Sigma-Aldrich, St. Louis, MO, USA), according to manufacturers’ instructions.

### 2.4. Animal Slaughter, Carcass Traits, and Tissue Sample Collection

After the experimental period, the steers were transported 226 km to a commercial packing facility (Plena Alimentos, Pará de Minas, MG). The animals were stunned by the concussion technique using the captive bolt and death by exsanguination of the jugular vein, followed by hide removal and evisceration. After, the carcasses were weighed to obtain the hot carcass weight and dressing percentage.

Twenty-four hours after cooling at 2 °C, the subcutaneous fat thickness was measured between the 12th and 13th ribs of the *longissimus thoracis* [31]. The ribeye area was also measured between the 12th and 13th ribs by outlining using transparency paper and then measuring using an LAI-3100 area meter (LI-COR, Lincoln, NE, USA). The marbling score was calculated by ether fat through equation proposed by Dow et al. [32]. And the ether extract was analyzed using the FoodScan Meat Analyser TM^®^ (FOSS, Hillerød, Denmark) with near-infrared spectrophotometer technology (AOAC analysis method: 2007-04).

### 2.5. Data Analyses

The data were analyzed in a completely randomized design, and the animals were considered the experimental units. Initial BW was used as a covariate for performance characteristics and feeding behavior. Shapiro-Wilk test was performed to assess data normality, and when data did not present normal distribution, they were transformed using PROC RANK of SAS (SAS Inst. Inc., Cary, NC, USA). Individual intake, ADG, feed efficiency, carcass traits, D-lactate, TNF-α, and haptoglobin were analyzed using the MIXED procedure of SAS (SAS Inst. Inc., Cary, NC, USA), with diet, breed, and the diet × breed interaction as fixed effects and animals as a random effect.

The least squares means (LSMEANS) statement was used to calculate the adjusted means for treatments. The Tukey test was used to determine the differences between the means of the treatments when the diet × breed interaction was significant. Statistical significance was declared at *p* ≤ 0.05, and tendencies were defined as 0.05 < *p* ≤ 0.10.

## 3. Results

Angus × Nellore steers had greater DMI (8.7% greater, *p* = 0.01) and variation in DMI (kg) than Nellore steers (Table 2). In addition, Angus × Nellore steers had a greater ADG (*p* = 0.02), and consequently 18 kg more of final body weight (*p* = 0.02) than Nellore steers. Besides, Angus × Nellore tended to have greater feed efficiency (*p* = 0.10). Concerning carcass traits, Angus × Nellore steers had 1.49 mm more of subcutaneous fat thickness (*p* < 0.01) and tended to have greater longissimus muscle area (*p* = 0.09); however, they had a lower dressing percentage than Nellore steers (54.3 and 57.1%, *p* < 0.01).

Regarding the diets, steers fed the WSCB diet had greater DMI (*p* < 0.01) and lower DMI variation (kg and %, *p* < 0.01) than steers fed the WSC diet. For that reason, animals fed WSCB tended to have greater (*p* = 0.07) ADG and final BW. However, the use of WSC diets increased dressing percentage.

Nellore steers had lower NDF intake (8% lower, *p* < 0.01), but spent approximately 51 min longer ruminating (*p* < 0.01) and had a rumination rate, on average, 35 min longer per kg of NDF (*p* < 0.01) than Angus × Nellore (Table 3). Although there was no breed effect on the time spent chewing, Nellore steers had greater chewing/DM (*p* < 0.01) and tended to have greater chewing/NDF (*p* = 0.06) compared to Angus × Nellore. Steers fed the WSCB diet spent 141 min longer chewing (*p* < 0.01), and, consequently, had on average, 12 min longer chewing per kg of DM compared to steers fed WSC (*p* < 0.01). Steers fed the WSCB diet spent 111 min longer ruminating (*p* < 0.01) and tended to spend more time eating (*p* = 0.07). In addition, the steers fed the WSCB diet had proximally 51% more NDF intake (*p* < 0.01) and tended to have a greater rumination rate of NDF (*p* = 0.09) than steers fed WSC diet. WSC diets resulted in lower passage rate (*p* = 0.05). Lastly, there was an interaction of breed × diet (*p* = 0.03) for the rumination rate of DM. Nellore steers fed the WSCB diet had, on average, a rumination rate 16 min longer per kg of DM than Nellore steers fed WSC or Angus × Nellore steers fed either WSC or WSCB.

Nellore steers tended to have a greater concentration of D-Lactate regardless of diet (*p* = 0.07) (Table 4). Regarding immune response markers, Angus × Nellore had greater concentration of haptoglobin (*p* < 0.01), being proximally 74% greater in the blood of the crossbred steers than in the Nellore steers. In addition, there was no effect (*p* > 0.10) of diet and breed on concentration of TNF-α.

## 4. Discussion

The study evaluated performance, carcass traits, feeding behavior, and humoral immune response in Nellore and Angus × Nellore steers fed whole shelled corn diets with or without fiber and finished in the feedlot.

Although Angus × Nellore steers had greater DMI and consequently, greater energy intake (proximally 7%), which resulted in greater subcutaneous fat, a higher marbling score was not achieved by this genetic group, contrary to the expectation of our hypothesis. An explanation for this result may be the low slaughter weight of the steers due to low ADG and DMI, regardless of diet and breed.

The low slaughter weight can also be explained by the use of individual pens and use of castrated animals that were not implanted. Animals housed in individual pens may be under greater stress, which causes a reduction in DMI and consequently decreases performance [33], even when fed high concentrate diets. In our study, it was necessary to use individual pens to evaluate DMI variation, feeding behavior, passage rate, and run a digestibility trial, which is not a part of this paper. Marques et al. [20], working with animals housed in collective pens, found that Nellore bulls had greater ADG when fed WSC or WSCB (1.21 kg/day and 1.57 kg/day, respectively) compared to the present study, (0.90 kg/day and 1.05 kg/day).

Additionally, despite the greater final BW of Angus × Nellore, they have lower carcass dressing percentage than Nellore. This result is confirmed in the literature and may be due to Taurus animals having a larger gastrointestinal tract than Nellore bulls [2,34].

The lower DMI in Nellore steers compared to Angus × Nellore can be due to a greater production of organic acids and a possible reduction in ruminal pH when feed whole shelled corn diets, since those animals had lower NDF intake and tended to have greater D-lactate. The fiber stimulates rumination and salivation, and ruminal motility, avoid ruminal pH drop, affecting the digesta passage and consequently nutrient intake [35]. Thus, with our results we show that, the fiber intake, even in WSCB diet, may not have been adequate to buffer the rumen pH.

The negative effect on feed intake was greater in the Nellore since previous studies show that Zebu cattle is more sensitive and develop acidosis problems more frequently when consuming high-concentrate diets [22,36], resulting in digestive disorders and consequently in a lower feed intake [37].

In this current study, although the D-lactate values indicate that the animals did not have acute acidosis, the Nellore and Angus × Nellore steers may have subacute ruminal acidosis (SARA). According to Nagaraja et al. [38], the normal concentration of lactate in animals with SARA is below 5 mM. Therefore, a reduction in the rumen pH is due to an imbalance between the production and the clearance of ruminal short-chain fatty acids [37,39].

Regarding diets, the lower DMI in the steers fed WSC may be because of the greater variation of DMI (49% more variation in animals fed WSC than WSCB). Schwartzkopf-Genswein et al. [40] reported that variation in DMI greater than 10% can negatively affect the performance of beef cattle in the feedlot because of larger variation in the daily ruminal pH. Therefore, despite this great DMI variation, the overall results from this variation is an average reduction in DMI and, consequently, ADG. Another explanation for this result may be related to the greater metabolizable energy of the WSC diet (Table 1), which likely regulated intake chemostatically [41]. Therefore, the steers needed to consume less feed to meet their nutritional requirements.

Besides, the lower DMI in the steers fed the WSC diet might also be explained by a reduction in ruminal pH and, consequently, greater absorption of short-chain fatty acids may have activated the chemoreceptors in the muscle wall of the rumen–reticulum, decreasing gastrointestinal tract motility and consequently the DMI [42]. Although ruminal pH was not measured in this study, Carvalho et al. [2] found that the rumen pH of animals fed whole shelled corn remained below 5.8 for 17 h, and according to Beauchemin et al. [43] subclinical acidosis occurs when ruminal pH remains below 5.8 for over 12 h.

In addition, adding bagasse increased the passage rate, which resulted in greater DMI for the animals fed WSCB diet. The positive effect of a small amount of forage on DMI is extensively reported in the literature [19,20,44]. Forage stimulates rumination [45] and increases saliva flow and ruminal motility [46], which, in turn, increases ruminal pH. However, the greatest gain in the use of sugarcane bagasse in WSC diets is to establish rumen normal functioning which will result in greater intake and performance.

In addition to the effect on the rumen environment, some nutrients or the energy and protein ratio of the diet can modulate immunological, metabolic, and inflammatory processes, situations in which nutritional status depletion and clinical complications occur [47], affecting the healthy animal and its performance. The innate immune response can be assessed using humoral markers, such as cytokines and acute phase proteins [48,49]. In the present study, TNF-α and haptoglobin, indicators of the initial development of the innate inflammatory immune response, were evaluated.

Angus × Nellore had a greater concentration of haptoglobin, showing that these animals had an inflammatory response, which may be due to the greater DMI, lower rumination rate of DM, and high starch intake daily, which caused the onset of SARA. This immune response is due to an increase in the rumen and hindgut absorption of lipopolysaccharides (LPS). LPS works as a molecular pattern associated with the pathogen and, after its recognition by the organism, promotes the release of pro-inflammatory cytokines, which induces the expression of acute-phase proteins [37]. During SARA, reduction in rumen pH increases lysis of gram-negative bacteria, which increases LPS and evokes an immune response in the bloodstream [50]. According to Cooke [16], acute-phase protein responses demand considerable body resources, increase maintenance requirements, and decrease nutrient intake, which negatively affects the performance of the animal. In addition, leukocyte recruitment takes place, related to energy expenditure [17]. Araujo et al. [51] found a negative correlation (r ≤ −0.50) between concentration of acute-phase protein and the ADG of beef cattle. However, in our study, although the Angus × Nellore steers displayed a greater blood haptoglobin concentration, it was not enough to reduce the performance of these animals.

The immune response is stimulated by the molecular patterns detected and the presence of acute-phase proteins in the immune system of animals, which subsequently stimulate the production of macrophage proinflammatory cytokines at the infection site [52]. Although the WSCB increased starch intake (5.07 and 4.10; *p* < 0.01), which could decrease rumen pH [39], the greater fiber concentration and the lower variation in DMI may have been enough to prevent a decline in ruminal pH, avoiding greater production of LPS and greater inflammatory response in this diet compared to the WSC. For the WSC diet, the lower DMI can also explain why the TNF-α concentration was not greater than in the WSCB diet, since it had less substrate to rumen fermentation.

## 5. Conclusions

The use of fiber sources in low amounts, such as sugarcane bagasse, is recommended in whole shelled corn diets, as it increases DMI and ADG without affecting feed efficiency or carcass traits. Although the Angus × Nellore steers have increased haptoglobin concentration due to the higher DMI, this effect was not enough to reduce performance of these animals feeding whole shelled corn diets.

## Figures and Tables

**Table 1:** Percentage of ingredients and chemical composition of experimental diets.

Ingredients %	Diet (% DM)	
	Whole Shelled Corn	Whole Shelled Corn and Bagasse
**Ingredient, %**		
Whole shelled corn	80.0	74.0
Sugarcane bagasse	-	6.0
Protein and mineral supplement ^1^	20.0	20.0
**Nutrient, DM**		
Crude protein, %	15.0	14.7
Neutral detergent fiber, %	15.2	19.0
Non-fiber carbohydrates, %	60.0	56.7
Starch, %	57.2	52.9
Ether extract, %	3.17	3.03
Metabolizable energy, Mcal/kg DM	3.00	2.65

^1^ Assurance levels per kilogram of product: crude protein (CP)—32.5%, neutral detergent fiber (NDF)—21.6%, Ca—45 g, Mg—7.5 g/kg, P—11 g/kg, Cu—104 mg/kg, Zn—344 mg/kg, Se—0.83 mg/kg, Vitamin A—30.500 UI/kg, Vitamin D—3800 UI/kg, Vitamin E—134 UI/kg, and Rumensin—140 mg/kg.

**Table 2:** Performance and carcass traits of Nellore and Angus × Nellore steers fed the whole shelled corn diet (WSC) or the whole shelled corn with sugarcane bagasse diet (WSCB) for 96 days of feedlot.

Item	Nellore		Angus × Nellore		SEM ^3^	*p-*Value		
	WSC ^1^	WSCB ^2^	WSC	WSCB		Breed	Diet	B*D
Initial BW, kg	344	350	358	359	9.10	0.21	0.71	0.75
Final BW, kg	425	437	451	474	9.84	0.02	0.09	0.23
DMI, kg/day	6.95	7.94	7.46	8.73	0.26	0.01	<0.01	0.59
DMI, % BW	1.60	1.83	1.69	1.89	0.04	0.07	<0.01	0.70
DMI variation, %	16.88	11.31	17.92	11.99	1.10	0.44	<0.01	0.86
DMI variation, kg	1.15	0.87	1.31	0.99	0.067	0.04	<0.01	0.83
ADG, kg/day	0.841	0.901	0.959	1.196	0.077	0.02	0.06	0.23
Gain:feed, kg/kg	0.121	0.114	0.127	0.136	0.008	0.10	0.86	0.32
Hot carcass weight, kg	251	247	244	250	4.48	0.59	0.73	0.29
*Longissimus* area, cm^2^	72.9	68.3	75.4	77.6	3.12	0.09	0.58	0.34
Subcutaneous fat, mm	3.41	3.16	4.87	4.68	0.21	<0.01	0.28	0.89
Marbling ^4^	348	379	361	355	21.5	0.80	0.56	0.38
Dressing percentage, %	57.8	56.4	54.9	53.7	0.54	<0.01	0.01	0.87

BW = body weight; DMI = dry matter intake; ADG = average daily gain. ^1^ Diet containing 80% whole shelled corn and 20% pelleted protein, mineral, and vitamin supplement. ^2^ Diet containing 74% whole shelled corn; 20% pelleted protein, mineral, and vitamin supplement; and 6% sugarcane bagasse. ^3^ Standard Error of Means. ^4^ Marbling score: 300 = slight, 400 = small, 500 = modest, 600 = moderate, 700 = slightly abundant.

**Table 3:** Feeding behavior of Nellore and Angus × Nellore steers fed the whole shelled corn diet (WSC) or the whole shelled corn with sugarcane bagasse diet (WSCB) on day 50 of the experiment.

Item	Nellore		Angus × Nellore		SEM ^3^	*p* Values		
	WSC ^1^	WSCB ^2^	WSC	WSCB		Breed	Diet	B*D
Time spent eating, min	119	136	130	173	16.9	0.16	0.07	0.45
Time spent ruminating, min	139	287	125	199	20.0	0.01	<0.01	0.07
Time spent chewing, min	258	423	255	372	20.6	0.32	<0.01	0.36
Dry matter								
Eating rate of DM, min/kg	17.6	17.3	17.3	20.2	2.22	0.57	0.59	0.43
Rumination rate of DM, min/kg	20.3 b	36.8 a	17.4 b	22.9 b	2.39	<0.01	<0.01	0.03
Chewing/DM, min/kg	38.0	54.0	34.7	43.1	3.43	0.01	<0.01	0.43
Neutral detergent fiber								
NDF intake, kg ^4^	1.06	1.58	1.13	1.73	0.052	0.04	<0.01	0.39
Eating rate of NDF, min/kg	117	87	114	103	12.8	0.64	0.11	0.43
Rumination rate of NDF, min/kg	135	185	114	116	16.4	<0.01	0.09	0.11
Chewing/NDF, min/kg	252	272	228	219	20.3	0.06	0.77	0.47
Passage rate, %/h	4.44	4.75	3.14	4.89	0.52	0.25	0.05	0.16

DM = dry matter; NDF = Neutral detergent fiber. ^1^ Diet containing 80% whole shelled corn and 20% pelleted protein, mineral, and vitamin supplement. ^2^ Diet containing 74% whole shelled corn; 20% pelleted protein, mineral, and vitamin supplement; and 6% sugarcane bagasse. ^3^ Standard Error of Means. ^4^ Intake during the behavior analysis period.

**Table 4:** D-lactate concentration and immune response of Nellore and Angus × Nellore steers fed the ground whole shelled corn diet (WSC) or whole shelled corn with sugarcane bagasse (WSCB) diets on day 45 of the experiment.

Item	Nellore		Angus × Nellore		SEM ^3^	*p-*Value		
	WSC ^1^	WSCB ^2^	GMI	GMIB		Breed	Diet	B*D
D-Lactate, mM	2.11	2.26	1.58	1.90	0.24	0.07	0.32	0.72
Haptoglobin, ug/mL	80.93	70.46	119.40	144.16	21.54	<0.01	0.71	0.36
TNF-α, ng/mL	4.40	4.61	4.55	4.68	0.43	0.79	0.69	0.93

TNF-α = tumor necrosis factor alpha; ^1^ Diet containing 80% whole shelled corn and 20% pelleted protein, mineral, and vitamin supplement; ^2^ Diet containing 74% whole shelled corn; 20% pelleted protein, mineral, and vitamin supplement; and 6% sugarcane bagasse. ^3^ Standard Error of Means.

## Data Availability

The datasets analyzed in the current study are available from the corresponding author on reasonable request.
